# Prevalence of work-related musculoskeletal disorders and its associated factors among traditional cloth weavers in Chencha district, Gamo zone, Ethiopia, *an ergonomic study*

**DOI:** 10.1371/journal.pone.0293542

**Published:** 2023-11-09

**Authors:** Desta Haftu, Haregewein Kerebih, Amare Terfe

**Affiliations:** 1 Department of Public Health, School of Public Health, College of Medicine and Health Sciences, Arba Minch University, Arba Minch, Ethiopia; 2 Department of Anatomy, Debre Markos University, Debre Markos, Ethiopia; 3 Department of Environmental Health Science, College of Medicine and Health Sciences, Arba Minch University, Arba Minch, Ethiopia; Czestochowa University of Technology: Politechnika Czestochowska, POLAND

## Abstract

Musculoskeletal disorders at work are common in the majority of African countries. Weaving is very important in Ethiopia for the national economy and for enhancing the country’s cultural values. However, the prevalence of musculoskeletal disorders in developing countries is significantly higher in common informal or small-scale enterprises such as weaving. Moreover, little is known about the prevalence and risk factors for musculoskeletal disorders in the weaving industry in Ethiopia. Therefore, the purpose of this study was to determine the prevalence of work-related musculoskeletal disorders and associated factors among traditional cloth weavers in Chencha district, Gamo zone, Ethiopia. A community-based cross-sectional study was conducted in Chencha district from November 1 to December 30, 2021, using a simple random sampling technique. A total of 420 traditional cloth weavers working in individual households were interviewed. Multivariable logistic regressions were performed on variables with p-values less than 0.025 in the bivariate analysis. Work-related musculoskeletal disorders had been reported in the previous 12 months by approximately 97 (46.9%) of respondents. Work-related musculoskeletal disorders were reported by 76.1% of weavers in the shoulder region, 64.2% in the low back, 56.3% in the neck, and 0.2% in the upper back. Working for more than 10 years (AOR = 5.05, 95%CI: 1.23, 20.77), working with prolonged sitting (AOR = 4.77, 95%CI: 1.37, 16.62), and job dissatisfaction (AOR = 2.41, 95%CI: 1.04, 5.55) were among the determinants of work-related musculoskeletal disorders. As a result, ergonomically oriented weaving workstations are required because the majority of musculoskeletal disorders are caused by poorly designed workstations.

## Introduction

Work-related musculoskeletal disorders (WRMSDs) are musculoskeletal disorders caused by occupational exposure. They can affect muscles, joints and tendons, ligaments, nerves, or localized body circulation systems that are aggravated primarily by work and the effects of the immediate environment. Most Musculoskeletal disorders (MSDs) develop over time and can be episodic or chronic, progressing from mild to severe disorders [1, 2]. MSDs are a group of degenerative and inflammatory conditions that affect the blood vessels, nerves, joints, ligaments, tendons, and muscles. MSDs are the leading cause of disability, impaired body functions, decreased work capabilities, and decreased quality of life, resulting in significant economic implications for the nation [3–6]. MSDs occur when the body structure is repeatedly abused and forced to endure a workload that the body is unable to tolerate [7, 8].

MSDs are the second greatest cause of years lost to disability, according to the Global Burden of Disease 2017 report, even though years of life lost are falling in Sub-Saharan Africa. Furthermore, as the Sub-Saharan population ages, the burden of MSD will continue to increase [9–11]. MSDs are less prioritized in low-middle income countries (LMICs), particularly in Ethiopia, due to a focus on more pressing and life-threatening health issues such as infectious diseases. According to a previous comprehensive review done in Africa, the prevalence of MSDs ranged from 13 to 92% in South Africa and Ghana, respectively [12]. Evidence suggests that genetic diversity; differences in social structure, economics, and other factors may all have a role in the reported difference in the prevalence of MSDs among Africans. Furthermore, musculoskeletal issues remain a major global health concern and a significant burden for LMICs such as Ethiopia, where health funding is already limited and devoted to life-threatening conditions. According to research, the prevalence of musculoskeletal pain among Ethiopia’s working population is between 35% and 75% [13–15].

The vast majority of work-related deaths, injuries, and illnesses are preventable. However, there are approximately 2.78 million work-related deaths worldwide each year. Moreover, work-related deaths accounted for 5% of all deaths worldwide [16]. Every day, it is estimated that over 7,500 people die as a result of occupational accidents. The number of fatal occupational accidents has slightly increased. The estimated number of non-fatal occupational accidents is 374 million, a significant increase from 2010. Asia was the largest contributor, accounting for roughly two-thirds of global work-related mortality, followed by Africa (11.8%) and Europe (11.7%) [17].

Weaving is one of the world’s oldest surviving crafts, and it is the second largest source of rural employment after agriculture. Weaving is a significant cottage industry in developing countries such as India, Pakistan, Bangladesh, Iran, Turkey, and China, where traditional weaving methods are still widely practiced. Weaving is a largely unorganized industry in developing countries [18]. Informality harms workers’ rights, including fundamental principles and rights at work, social protection, and adequate working conditions [19]. Weaving involves a variety of activities that involve repetitive movement of upper and lower limbs to operate pedals and shuttles, with arms raised away from the body. As a result, weaving and other handloom activities have become high-risk occupations for developing musculoskeletal disorders [20]. MSDs are painful conditions caused by overuse of the body’s muscles, joints, nerves, tendons, and soft tissues. Because of the impact on worker health and productivity, MSDs are considered one of the most expensive occupational disorders [21]. Furthermore, previous studies on weavers have revealed a widespread prevalence of MSD among workers, which is mostly attributable to working conditions and exacerbated by work-related stress [22].

A variety of factors influence the occurrence of MSDs in various professions. Working postures are influenced by the shape, arrangement, size, and placement of the tools used as well as their operating methods. Unnatural body positions and non-ergonomic ways of working for an extended period can result in a variety of health issues for employees. Working in the same position for an extended period whether standing or sitting will cause discomfort [23]. Working postures such as sitting for an extended time without any adjustment can soften the abdominal muscles, causing spine curvature and causing respiratory and digestive organ disorders [24]. Furthermore, a poor ergonomic workstation has been shown to increase muscular load and activity, increasing MSD [25]. Physical factors or loads on the biomechanical system are thought to cause tissue damage and inflammation, leading to MSD [26]. However, there is still conflicting evidence that there is a link between workstation configuration and MSD [27, 28].

Work-related MSD problems are prevalent. They reduce the productivity and revenue of many Ethiopian weavers. Managing MSD provides for increased productivity and improved quality of life. Despite substantial studies on the effects of MSD on other occupations and the need to treat them, there is limited data on the weaving industry, and no equivalent research has been conducted. There are no published articles in this field in Ethiopia, there is a research deficit and a need for an integrated approach to MSD prevention. Weavers in this study may be able to close a knowledge gap by giving recorded proof of MSD prevalence and advocating for preventative steps to mitigate its impact. Moreover, Weaving provides a source of income and employment opportunities not only for weavers and their families, but also for traders, tailors, fashion designers, and soon. Weaving is very important in Ethiopia for the national economy and for enhancing the country’s cultural values [29]. Furthermore, musculoskeletal disorders continue to be a major global health issue as well as a significant financial burden for low- and middle-income countries such as Ethiopia, where health budgets are already stretched thin and devoted to life-threatening conditions. According to one systematic review, musculoskeletal pain affects anywhere from 35% to 74.5% of Ethiopia’s working population [30].

For centuries, Ethiopians have practiced traditional cotton weaving using both endogenous and exogenous technology. Cotton weavers work in small, cramped spaces under appalling working conditions. People working in the weaving industry are extremely vulnerable due to a lack of occupational safety and health services and poor working conditions because they are not supported by occupational safety and health services [29]. Even in modern times, weaving has cultural value, and weavers must be properly cared for and valued as artisans. This research is critical not only for the health of weavers but also for the aesthetic and cultural value of the weaving profession. Traditional cloth weaving is still practiced in many parts of Ethiopia. The occupation has contributed significantly to the economies of both the workers in the sector and the country as a whole. There is, however, a scarcity of information about the health issues that weavers face at work. As a result, the purpose of this study was to ascertain the prevalence of work-related musculoskeletal disorders and their associated factors among traditional cloth weavers in the Chencha district.

## Methods

### Study setting and design

Community—based cross-sectional study design was conducted from November 1, 2021, to December 30, 2021, GC. In Ethiopia, weaving production is one of the informal sectors, and most weavers work from home and are self-employed. According to the 2002 Central Statistical Agency (CSA) report, there are more than 221,848 weavers in Ethiopia, and 45% of the weavers lived in cities, while the remaining 55% lived in rural areas. Dorze people in the Chencha district, Gamo zone, were well-known for their woven goods. In Ethiopia, Gamo is the source of weaving knowledge. The majority of the weavers migrated from various rural areas of the Region over the years [31]. Chencha is 250 kilometers south of the capital of the southern regional state, Hawassa; and 480 km southeast of the capital city of Ethiopia, Addis Ababa.

### Sample size

This study included all traditional cloth weavers in the Chencha district who work in individual households and meet the following inclusion criteria: weavers with more than one year of work experience and weavers who work weaving full-time. The sample size was determined using a single population proportion formula by considering 50% prevalence since we could not get a study conducted in Ethiopia and other similar countries in Africa on this topic, a 95% level of confidence, and a 5% margin of error. By adding a 10% non-response rate, the final sample size was 423 traditional cloth weavers.

### Sampling procedures

This study included all traditional cloth weavers from the Chencha district who work in individual households. The study subjects were weavers with more than one year of experience to determine the 12-month prevalence of low back pain among weavers. This study included 423 traditional cloth weavers from randomly selected 7 clusters which represent 50% of the district clusters which were selected using cluster-level randomization. According to the Chencha district report, there are more than 18, 278 weavers in the district. The clusters were identified using an Ethiopian population census enumeration area map. Cluster sampling was used to choose representative sample clusters within the Chencha district to draw the sample of households with weavers using a simple random sampling method. The households in the selected clusters with at least one traditional cloth weaver were then censused and coded. Finally, households were chosen at random. If a home had more than one weaver (head of household), the lottery method was used to select one weaver. The weavers were recruited when they were volunteers and provided consent to participate in this study before the data collection process.

### Data collection procedures and quality assurance

The final questionnaire included two domains: one to collect the prevalence of WMSD using the Nordic Musculoskeletal-Extended (NMQ-E) and another to collect associated factors. The prevalence data were collected using the Standardized Nordic Musculoskeletal-Extended (NMQ-E) questionnaires for assessing the prevalence of work-related musculoskeletal disorders [32] by face-to-face interviews from traditional cloth weavers who work in individual households in the Chencha district. In addition, an observational checklist was filled out by direct observation to collect additional information on MSDs. The other questionnaire was created by reviewing various relevant literature to obtain information on determinants of musculoskeletal disorders such as sociodemographic characteristics, environmental factors, organizational factors, behavioral factors, and psychosocial factors.

A training manual was created to aid in the training process for data quality assurance. The training was primarily focused on interviewing techniques, with an emphasis on questions that require close attention and observation. The training included a classroom lecture, a mock interview, and field practice. The supervisors were in charge of supervising the data collectors, checking for completed questionnaires, and correcting any errors or problems that arose. The principal investigators oversaw the entire data collection process. Finally, 5% of the households were re-interviewed by supervisors/principal investigators to ensure consistency of data collection, and corrections will be made on the spot.

### Operational definitions

#### Work-related musculoskeletal disorder

Is perceived pain, ache, discomfort, stiffness, or fatigue for at least 2–3 work days in the last week or last 12 months in any part of body segments (neck, shoulder, upper back, lower back) of the weavers that are being caused by primarily the performance of work, aggravated or exacerbated by the effects of the immediate environment in which work is carried out [33].

#### Stress

Workers who scored a mean of 12.5 and below were considered to have job stress using five job stress questions and 4 points Likert scale [34].

#### Job satisfaction

Workers who have scored a mean of 15 and above was considered to be satisfied and below 15 will be considered to be dissatisfied with his/her job using 5 points Likert scale [35].

### Variables

#### Independent variables

Socio-demographic factors

Age, sex, educational status, wealth index, work experience, BMI
Working condition factors
Working hours, working days, prolonged bending, prolonged sitting, static posture and repetitive body movement

Environmental factors

Presence of back support, presence of work place supervision, presence of health and safety training and work station design

Behavioral factors

Taking break(resting time), physical exercise or sporting activities, knowledge

Psychological factors

Stress, fatigue, job satisfaction

### Dependent variable

Work related musculoskeletal disorders

### Data management and analysis procedures

The investigator also checked the collected data for completeness and consistency. The data was cleaned, coded, and entered into EPI-Info 3.5.4 before being exported to STATA Version 15 for further analysis. Tables, figures, and charts were used to compute and describe descriptive statistics. The strength of the association between MSDs and associated factors was calculated using odds ratio (OR) and 95% confidence intervals to determine the independent effect of the independent variables with the dependent variable. Bivariate analysis was used to estimate crude OR, then multivariable logistic regression analysis was used to estimate adjusted OR with respective 95% CI for variables that were significant in the bivariate analysis. Statistical significance was defined as a P value less than 0.05 (5%). The Hosmer Lemeshow statistic was used to assess the model’s goodness of fit by determining whether the necessary assumptions for the application of multivariable logistic regression were met.

### Ethical consideration

The Arba Minch University Institution Review Board (IRB) provided ethical approval and clearance with approval number IRB/141/12, and the Chencha district administrative office provided permission. Furthermore, each study participant provided informed verbal consent. Respondents were informed that they had the option to skip or stop the interview process at any time. Furthermore, study subjects were informed that the information obtained from them would be kept confidential and used solely for the research. Finally, those with WMSD received health education and were linked to health institutions for assistance. For linking the weavers with WRMSDs to healthcare facilities, only the cross-ponding author had access to information about the participants.

## Result

### Socio-demographic characteristics of the respondent

A total of 420 traditional cloth weavers were included in the study with a response rate of 99.29%. Four hundred and nineteen (99.8%) respondents were male weavers. Approximately 33.3% of the weavers had the education level of primary school and only (0.7%) of traditional cloth weavers had the educational level of university/college. Approximately 145 (34.5%) respondents were in the older age group (greater than 40 years of age) and the median age of the study subject was 30 ± 13.21 SD. The traditional cloth weavers’ work experience revealed that 198 (47.1%) had served for more than 10 years. In terms of monthly income, 196 (46.7%) weavers had a monthly income of less than 1950 Ethiopian Birr (< 2.15 U$D per person per day) with a median of 2000 Ethiopian Birr (Table 1). Those weavers are classified as poor by the WHO global poverty line.

**Table 1:** Socio-demographic characteristics of traditional cloth weavers in Chencha district, Gamo Zone, Ethiopia, 2022.

Variables		Frequency	Percent (%)
Sex	Male	419	99.8
	Female	1	0.2
Age	< 20	90	21.4
	20–24	36	8.6
	25–29	60	14.3
	30–34	48	11.4
	35–39	41	9.8
	>40	145	34.5
Educational status	Illiterate	136	32.4
	Primary school(1–6)	139	33.1
	High school(7–10)	129	30.7
	Preparatory (11–12)	13	3.1
	University/College	3	0.7
Monthly income	≤ 1950 birr (≤ 1.25 USD)	196	46.7
	> 1950 birr (> 1.25 USD)	224	53.3
Work experience	1–5 years	118	28.1
	5–10 years	104	24.8
	˃ 10 years	198	47.1

### Prevalence of WRMSDs among traditional Weavers

Among 420(100%) respondents, 204(48.6%) of them reported WRMSDs after beginning weaving (95%CI: 44.1%, 53.6%). Approximately 197(46.9%) of the respondents with WRMSDs through their job career had WRMSDs during the last 12 months (95%CI: 41.7%, 51.9%). Among those weavers who reported WRMSDs in the last 12 months, 174(41.4%) of them felt WRMSDs during the previous seven days (Table 2).

**Table 2:** Prevalence and characteristics of WRMSDs among traditional cloth weavers in Chencha district, Gamo Zone, Ethiopia, 2022.

Variables		Number	Percent
History of WRMSDs	Yes	31	7.4
	No	389	92.6
Body parts affected by a history of WRMSDS	Neck	21	5.0
	Low back	10	2.4
WRMSDs-related pain after starting weaving	Yes	204	48.6
	No	216	51.4
Body parts affected pain after starting weaving	Neck	57	13.6
	Shoulder	63	15.0
	Low back	83	19.8
	Upper back	1	0.2
WRMSDs in the last 12 months	Yes	197	46.9
	No	223	53.1
Body parts affected due to WRMSDs in the last 12 months	Neck	56	13.3
	Shoulder	76	18.1
	Low back	64	15.2
	Upper back	1	0.2
Duration of the WRMS pain	1 Day	1	0.2
	2–7 Days	117	27.9
	More than a week	75	17.9
	Do not recall	4	1.0
WRMSDs in the last 7 days	Yes	174	41.4
	No	246	58.6
Body parts affected due to WRMSDs in the last 7 days	Neck	31	7.4
	Shoulder	43	10.2
	Low back	109	26.0
	Upper back	1	0.2
Hospitalization due to WRMSDs	Yes	101	24.0
	No	319	76.0
Causes of hospitalization	Neck pain	38	9
	Shoulder pain	39	9.3
	Low back pain	23	5.5
	Upper back pain	1	0.2
Frequency of Hospitalization due to WRMSDs	Once	59	14
	More than once	42	10
Changing jobs due to WRMSDs	Yes	17	4
	No	403	96
Reducing activity due to WRMSDs	Yes	65	15.5
	No	355	84.5
Absent from work due to WRMSDs	Yes	118	53.1
	No	79	28.1
Duration absent from work due to WRMSDs	0 Day	302	71.9
	1–7 Days	12	2.9
	8–30 Days	48	11.4
	More than 30 days	58	13.8

The number of hospitalizations for WRMSDs in the previous 12 months was 101(24%). Out of those cases, 39(9.3%) of them were hospitalized due to shoulder pain and 42(10%) weavers were hospitalized more than once during the previous 12 months. In terms of work absenteeism, 118(28.1%) of the respondents were absent due to WRMSDs and among them 102(48.1%), 58(13.8%) were absent for more than 30 working days consecutively during the previous 12 months (Table 2).

### Determinants of WRMSDs

Bivariate and multivariate logistic regression models were used to analyze socio-demographic, working conditions, and environmental, behavioral, and psychosocial determinants (factors). In the bivariate logistic regression analysis, working in an uncomfortable posture, working in the same posture for an extended period, making the same body movement, taking breaks, and resting time all had a statistically significant association with WRMSDs. WRMSDs were significantly associated with those weavers who had more than 10 years of work experience (AOR 5.05, 95%CI 1.23, 20.77) and those weavers who had a history of WRMSDs (AOR 8.34, 95%CI 2.23, 31.26) in the multivariate logistic regression analysis (Table 3).

**Table 3:** Determinants of WRMSDs among traditional cloth weavers in Chencha district, Gamo Zone, Ethiopia, 2022.

Variables		WRMSDs		COR (95% CI)	AOR (95% CI)	P value
		Yes	No			
Age	< 20	32	58	1	1	
	20–24	15	21	0.77(0.35,1.70)	1.07(0.34,3.36)	0.90
	25–29	34	26	0.42(0.21,0.82)	0.91(0.26,3.13)	0.88
	30–34	26	22	0.47(0.23,0.95)	2.01(0.49,8.34)	0.33
	35–39	18	23	0.71(0.33,1.49)	0.79(0.17,3.72)	0.77
	> 40	72	73	0.56(0.33,0.96)	1.67(0.39,7.15)	0.49
Work experience	< 5 years	103	92	1	1	
	5–10 years	54	50	1.00(0.62,1.61)	2.02(0.67,6.09)	0.21
	>10 years	40	78	**2.11(1.31,3.39)**	**5.05(1.23,20.77)**	**0.025**
History of WRMSDs	Yes	26	5	**6.63(2.49,17.63)**	**8.34(2.23,31.26)**	**0.002**
	No	171	218	1	1	
Working hours per day	< = 8 hours	182	161	1	1	
	>8 hours	15	62	**4.67(2.56,8.54)** ***	2.16(0.93,5.03)	0.07
Working in a Chair with no back support	Yes	53	136	**4.25(2.81,6.43)** ***	2.00(0.77,5.24)	0.16
	No	144	87	1	1	
Working with sitting for a prolonged time	Yes	28	104	**5.28(3.27,8.51)** ***	**4.77(1.37,16.62)**	**0.01**
	No	169	119	1	1	
Working in an uncomfortable posture	Yes	108	170	**2.64(1.74,4.01)** ***	1.67(0.82,3.51)	0.15
	No	89	53	1	1	
Working in the same posture for a prolonged time	Yes	68	151	**3.97(2.65,5.97)** ***	2.53(0.70,9.11)	0.16
	No	129	72	1	1	
Making the same body movement	Yes	57	87	**1.57(1.04,2.37)** **	0.39(0.11,1.39)	0.15
	No	140	136	1	1	
Taking break	Yes	191	160	1	1	
	No	6	63	**12.53(5.29,29.72)** ***	0.42(0.10.1.67)	0.22
Resting time	1 hour	126	64	1	1	
	2 hours	13	111	**16.81(8.79,32.15)** ***	1.88(0.59,5.96)	0.28
	3 hours	25	27	**2.13(1.14,3.96)** *	0.23(0.05,1.03)	0.06
	>4 hours	33	11	1.25(0.67,2.34)	0.37(0.08,1.78)	0.22
Regular physical exercise	Yes	143	125	1	1	
	No	54	98	**2.08(1.38,3.13)** ***	**2.88(1.43,5.79)**	**0.003**
Knowledge of causes of WRMSDs	Yes	165	69	1	1	
	No	32	154	**11.51(7.17,18.47)** ***	**8.08(3.76,17.35)**	**0.0001**
Job Satisfaction	Satisfied	17	47	1	**1**	
	Not satisfied	180	176	**2.83(1.56,5.11)** **	**2.41(1.04,5.55)**	**0.04**

Note: significant at, * *p* ≤ 0.05

** *p* ≤ 0.01

*** *p* ≤ 0.001, COR = Crude Odds Ratio and AOR = Adjusted Odds Ratio

## Discussion

Weaving is one of the most taxing occupations, with long hours of static work that leads to Musculoskeletal Disorders. WRMSDs were extremely common among weavers working in small-scale industries and the informal sector. Furthermore, socio-demographic factors, working conditions, and weavers’ behavioral and psychosocial characteristics were among the factors that contributed to the occurrence of WRMSDs [18, 36–38]. This Study determined the prevalence of WRMSDs and associated factors among traditional cloth weavers in Chencha, Gamo Zone, Ethiopia.

According to the findings of this study, the prevalence of WRMSDS among traditional cloth weavers was 204 (48.6%) after beginning their weaving career (95% CI: 43.6%, 53.6%). Among study participants, the 12-month prevalence was 197 (46.9%) (95% CI: 41.9%, 51.9%). The prevalence of WRMSDs in this study, however, is lower than in the study conducted in Varanasi, India. The Indian study found that MSDs were more common in different parts of the body in the previous year among handloom and power loom workers. According to the study, 96.13% of weavers, including both handloom and power loom workers, reported having a problem in different body regions [39]. The difference in the prevalence of WRMSDs could be due to differences in the working conditions of weavers in these two studies. In India, the study was conducted in well-organized workshops with a poor working environment, whereas this study was conducted in individual households with less organized working shops. Furthermore, there are differences in working conditions between handloom, power loom, and traditional cloth weaving.

Weavers with WRMSDs in the shoulder 76(18.1%), low back 64(15.2%), neck 56(13.3%), and upper back 1(0.2%). This study found that the shoulder is the most common WRMSD among traditional cloth weavers, which is similar to a study done among handloom weavers that found the shoulder to be the most prevalent region [36, 38]. However, this study contradicts the study done in India which reported that low back pain was the most common WRMSD among handloom weavers [37]. The possible difference may be the working condition in traditional cloth and handloom weaving is different. The Indian study focused on well-organized workshops with a poor working environment, whereas this study focused on individual households with less organized workshops. Furthermore, working conditions differ between handloom in India and traditional cloth weaving in Ethiopia.

In this study, weavers with a history of WRMSDs were more likely to develop WRMSDs. Weavers who had a history of WRMSDs were 8.34 times more likely to develop WRMSDs than those who had no history (AOR = 8.34, 95%CI: 2.23, 31.26). This study was consistent with a previous study conducted among healthcare workers and garment industry workers, which reported that participants with a history of musculoskeletal diseases were 6.585 and 6.6 times more likely to develop work-related musculoskeletal diseases than those without a history of MSD, respectively [40, 41]. This could be because when weavers are engaged in similar tasks, this could potentially aggravate the previous history of MSDs and also result in complicated health problems.

Weavers with more than ten years of experience were 5.05 times more likely to have WRMSDs than those with less than five years of experience (AOR = 5.05, 95%CI: 1.23,20.77). The findings are similar to those of a study among handloom weavers, which reported that working for more than ten years was a risk factor for WRMSDs [33, 39, 42]. The possibility is that weavers with more experience work for longer periods and are exposed to factors that may contribute to the occurrence of WRMSDs. Moreover, possible causes could also include aging, age-related degenerative changes, decreased tissue repair, cartilage thinning, and cumulative stress to various body structures as a result of workload. In contrast, an Indonesian study reported that more work experience significantly reduced the prevalence of WRMSDs [22]. The possible reason for the difference could be those Indonesian women weavers tend to take more rest, had reduced workloads, and improved working conditions compared to the weavers in this study.

Working hours per day, on the other hand, did not show a significant association with WRMSDs in this study. This study was consistent with studies conducted among handloom workers in India, Indonesia, and Bangladesh garment workers [43, 44]. The reason for this could be that daily working time in the weaving industry is not usually fixed; it varies depending on the situation and workload, and weavers’ motivation to earn more money causes them to work longer. However, this study contradicted studies conducted in Assam, Northern India, and Indonesia which found that long working hours affect the health of weavers who must spend a day weaving in looms. According to worker responses, as working hours increase, so does the health burden [42, 45]. The possible difference could be the weavers in this study are not formal sector weavers like the weavers in the North Indian and Indonesian studies which gives them the mandate to determine the working hours by themselves depending on whether they are feeling discomfort or not. The informality of the weavers in this study could have contributed to those weavers working less and suffering from MSDs.

Studies in various careers/professions revealed that working with prolonged sitting contributes to the occurrence of musculoskeletal problems in various body regions [22, 40, 42, 46]. Similarly, this study found that weavers who sat for extended periods were approximately 4.77 times more likely to report WRMSDs than those who did not sit for extended periods (AOR = 4.77, 95%CI: 1.37,16.62). The outcome is not surprising given that weavers used to weave for long periods while seated, with a stretched, extended body and repeatable movement [18]. The possible reason might be weavers sitting on an uncomfortable chair and table tends to develop stiffness and discomfort. This study was similar to a study conducted in Indonesia [22]. This is because the longer the working duration, the greater the exposure to risk factors. Long-term physical stress reduces muscle performance in areas with less mobility. The stresses will build up day by day for a long period, causing health concerns. However, this study contradicted a study conducted among carpet weavers in Iran, which found that prolonged sitting was not significantly associated with WRMSDs [27].

Another variable that was associated with WRMSDs was regular physical exercise. Weavers who did not engage in regular physical activities were 2.88 times more likely to develop WRMSDs than those who engaged in regular physical activities (AOR = 2.88, 95%CI: 1.43, 5.79). This study agreed with a systematic review of the effects of exercise on musculoskeletal disorders and a study done among school teachers in Ethiopia [13, 46]. The possible reasons may be exercise can enhance strength, flexibility, and pain tolerance, and make muscles and ligaments stronger to support various body parts without feeling discomfort.

Finally, this study found that weavers who were dissatisfied with their jobs were 2.41 times more likely to develop WRMSDs than weavers who were satisfied with their jobs (AOR = 2.41, 95%CI: 1.04, 5.55). This study was in line with studies conducted in Varanasi, India among weavers and emergency nurses in Iran, which reported job dissatisfaction increases the odds of having WRMSDs which could be contributed to physical and mental discomfort attributed to the working conditions in those professions [39, 47]. However, the result of this study contradicts studies done among nurses in Ethiopia [15]. The discrepancy could be related to the physical demands of weaving and nursing. Furthermore, it could be related to the disparity in earnings and working conditions between weaving and nursing jobs.

### Limitations

The cross-sectional nature of the data makes determining cause and effect impossible. As a result, because this study only included weavers who work in households, the findings should be interpreted with caution. Future research should address these concerns and assess the cause and effect among weavers working in both the formal and informal sectors. Furthermore, we did not do postural analysis with ergonomics tools. Nonetheless, this is a preliminary study to provide a powered insight into the prevalence of MSDs among Ethiopian weavers.

## Conclusions and recommendations

This study discovered a high prevalence of WRMSDs among traditional cloth weavers, indicating the need for immediate public health intervention. WRMSDs were also linked to work experience, working with prolonged sitting, a history of musculoskeletal disorders, regular physical activity, and job satisfaction. The study implies that there must be measures aimed at the prevention and control of MSDs, such as the provision of health and safety training and a safe working environment for weavers. Moreover, ergonomically oriented weaving workstations are required because the majority of WRMSDs are caused by poorly designed workstations. As a result, even minor changes in working conditions, weaving tool design, and working methods can result in significant benefits, such as reduced bending, uncomfortable posture, and tasks that put a strain on weavers’ backs. Therefore, concerned stakeholders should intervene to prevent the occurrence of WRMSDs among weavers and to improve the productivity of the sector using the finding of this study. Other scholars could also conduct interventional studies that could potentially solve the MSD problem faced by those weavers due to different factors, particularly workstation-related factors.
